# The spinal cord injury (SCI) peer support evaluation tool: the development of a tool to assess outcomes of peer support programs within SCI community-based organizations

**DOI:** 10.1038/s41393-024-01033-1

**Published:** 2024-09-23

**Authors:** Shane N. Sweet, Zhiyang Shi, Olivia Pastore, Robert B. Shaw, Jacques Comeau, Heather L. Gainforth, Christopher B. McBride, Vanessa K. Noonan, Launel Scott, Haley Flaro, Sheila Casemore, Lubna Aslam, Teren Clarke, Kathleen A. Martin Ginis

**Affiliations:** 1 Department of Kinesiology and Physical Education, McGill University, Montreal, QC Canada; 2 Centre for Interdisciplinary Rehabilitation Research in Metropolitan Montreal, Montreal, QC Canada; 3 School of Health & Exercise Sciences, University of British Columbia, Kelowna, BC Canada; 4 International Collaboration on Repair Discoveries (ICORD), University of British Columbia, Vancouver, BC Canada; 5 Spinal Cord Injury BC, Vancouver, BC Canada; 6 Praxis Spinal Cord Institute, Vancouver, BC Canada; 7 Spinal Cord Injury Saskatchewan Inc., Saskatoon, SK Canada; 8 Ability New Brunswick, Fredericton, NB Canada; 9 Spinal Cord Injury Ontario, Toronto, ON Canada; 10 Spinal Cord Injury Alberta, Calgary AB, Canada; 11 Department of Medicine, University of British Columbia, Vancouver, BC Canada; 12 Centre for Chronic Disease Prevention and Management, Faculty of Medicine, University of British Columbia, Kelowna, BC Canada

**Keywords:** Patient education, Quality of life

## Abstract

**Study design:**

Guided by the 4-step process outlined in the Consensus-based Standards for the selection of health Measurement INstruments (COSMIN) guideline, multiple methodologies were used: Delphi, literature reviews, ratings with consensus, think-aloud, and test-retest.

**Objectives:**

The purpose of this study was to develop and test a spinal cord injury (SCI) peer support evaluation tool that meets the needs of community-based SCI organizations in Canada.

**Setting:**

Peer support programs for people with SCI delivered by community-based SCI organizations.

**Methods:**

This research was co-constructed with executives and staff from SCI community-based organizations, people with SCI, researchers, and students. Given the multiple steps of this study, sample size and characteristics varied based on each step. Participants included people with SCI who received peer support (mentees) or provided peer support (mentors/supporters) and staff of community-based organizations.

**Results:**

In step 1, the 20 most important outcomes for SCI peer support were identified. In step 2 and 3, the 97 items were identified to assess the outcomes and by using rating and multiple consensus methodologies 20 items, one to assess each outcome, were selected. In step 4, content and face validity and test-retest reliability were achieved. The resulting SCI Peer Support Evaluation Tool consists of 20 single-item questions to assess 20 outcomes of SCI peer support.

**Conclusion:**

Through a systematic process, the SCI Peer Support Evaluation Tool is now ready to be implemented to assess outcomes of SCI peer support programs delivered by community-based SCI organizations.

## Introduction

Over 86,000 individuals in Canada have a spinal cord injury (SCI), with approximately 4000 new cases per annum [1]. Long-term health complications of a SCI (e.g., pressure injuries) place strain on the healthcare system and public/government services as impaired physical functioning can complicate obtaining or returning to employment [2]. The complex interaction of secondary health conditions and physical impairment associated with SCI can also alter social relationships and social participation opportunities [3–5]. These challenges contribute to the estimated $2.67 billion annual economic investment associated with SCI [6].

Programs and public services that help individuals with SCI manage and overcome the challenges of living with SCI play an important role in improving quality of life and reducing the strain on public health care and government services. Peer support programs are a widely available service in Canada, defined by our team as a peer interaction that aims to help individuals who share similar lived experiences adapt and/or thrive. Peer support programs offered by Canadian community-based organizations primarily adopt a discussion-based approach by facilitating one-on-one or group discussions between individuals with SCI [7]. Peer support has the potential to improve overall well-being and quality of life of people with SCI [8]. While peer support is recognized as an important service across Canada [7], it currently lacks high quality evidence of its effectiveness [7, 8]. This lack of evidence is partly attributable to the difficulties in aggregating data from different SCI peer support programs [7] due to inconsistent outcome reporting and variation of measurement instruments [8], as well as different focuses of the programs [7]. The difficulty in evaluating peer support programs delivered by community-based organizations is particularly relevant for Canadian SCI research. Research-based trials are difficult to implement in Canadian community settings, making the synthesis of data from community SCI peer support programs important for assessing evidence of effectiveness.

Development of a SCI peer support specific outcome measurement instrument (OMI; defined as a tool to measure quality or quantity of outcomes) [9] using a core outcome set would benefit SCI researchers and community program providers. A *core outcome set* is defined as a set of important outcomes, agreed upon by consensus, that should be measured to evaluate the effectiveness of programs for a specific population (e.g., persons with SCI) and context (e.g., peer support) [10]. No universal SCI peer support measure or core outcome set currently exists. There is therefore an inherent need to develop a SCI peer support OMI. This OMI can therefore help (1) researchers, (2) community organizations, and (3) partnership between researchers and community organizations to collect data using consistent outcome measures, that can be compared and aggregated across studies to determine the effectiveness of SCI peer support programs. Therefore, the purpose of this study was to develop and test a spinal cord injury (SCI) peer support evaluation tool by following and adapting the COSMIN guideline for community-based settings.

## Methods

### Design

This study used and adapted the four-step process to select core outcomes set by the Outcome Measures in Effectiveness Trials (COMET) initiative and the Consensus-based Standards for the selection of health Measurement INstruments (COSMIN) initiative [9]. The four-step process has been used to develop OMIs for various disability groups [11] and for programs occurring in community settings [12]. Importantly, according to the COSMIN guideline, OMIs are not homogenous in their structure and can include various measurement approaches (e.g., single item measures, questionnaires, a score obtained through physical examination, a laboratory measurement, etc.) [9]. These steps allowed for a methodological process to identify and select outcomes that would be important for a community-based evaluation tool for SCI peer support programs. Appropriate methodologies were used for each step: Delphi consensus, measurement literature review, quality rating and community consensus methods, think-aloud, and test-retest. Ethic certificates were obtained for steps where participant data collection was conducted (McGill REB file #: 21-11-011). Participants provided informed consent before data collection.

This work was conducted by a community-university partnership. We used the integrated knowledge translation guiding principles for SCI research to guide this partnership [13]. The roles of each team member across research phases are presented in Supplemental 1.

### Procedures

#### Step 1. Conceptual considerations

**Fig. 1:**
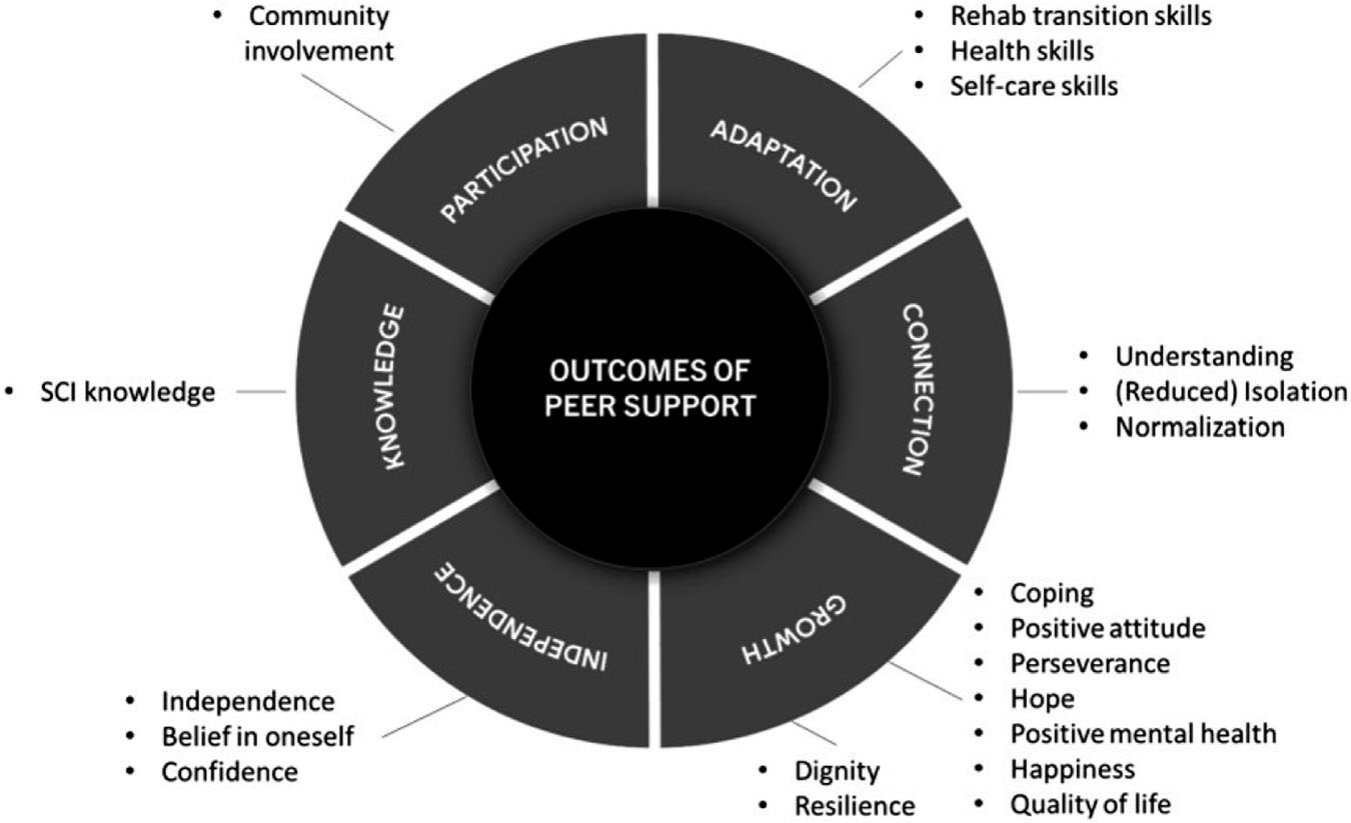
Outcomes identified and selected organized by a peer support outcome categorization. Note. Figure adapted from Rocchi et al. [14], reprinted by permission of the publisher (Taylor & Francis Ltd, http://www.tandfonline.com).

Prior to selecting outcomes of SCI peer support programs delivered by community-based organizations, an understanding of the relevant outcomes to this context was needed. Published in two separate papers, our group identified 87 outcomes relevant to SCI peer support through meta-synthesis and a qualitative study among peer support users [14, 15] (87 outcomes were listed in Supplemental 6). The peer support outcome model developed from the meta-synthesis also guided this study to ensure that each category was represented by at least one outcome (Fig. 1). We conducted two Delphi consensus studies among peer support users (Delphi 1) and peer support program coordinators and directors (Delphi 2) to identify the most important outcomes for SCI peer support. Detailed information on the Delphi methodology is available in our previous publication [16].

#### Step 2. Finding existing outcome measurement instruments

To reduce burden on peer support users/peer mentees who will be completing the core outcome set (hereafter referred to as the SCI Peer Support Evaluation tool), our partnership decided to use a single-item measure for each outcome. This decision aligns with a recent call for single-item measures in time-restricted conditions to facilitate response and to reduce data-processing costs [17], which are important for community-based organizations. Regarding outcomes identified in Step 1, online databases (e.g., Neuro-QOL, NIH Toolbox, PROMIS, ASCQ-Me, PsychINFO, MEDline) were individually searched by two researchers (ZS, OP) for validated single-item and multi-item measures for each outcome (see Supplemental 2). ZS and SS searched and identified items that related to the outcomes from surveys of our partnered community-based organizations. For each outcome, ZS and OP each selected validated multi-item and single-item measures that aligned with the outcome definitions. Outcome definitions were informed by the data from the meta-synthesis [14].

#### Step 3. Quality assessment of outcome measurement instruments

**Fig. 2:**
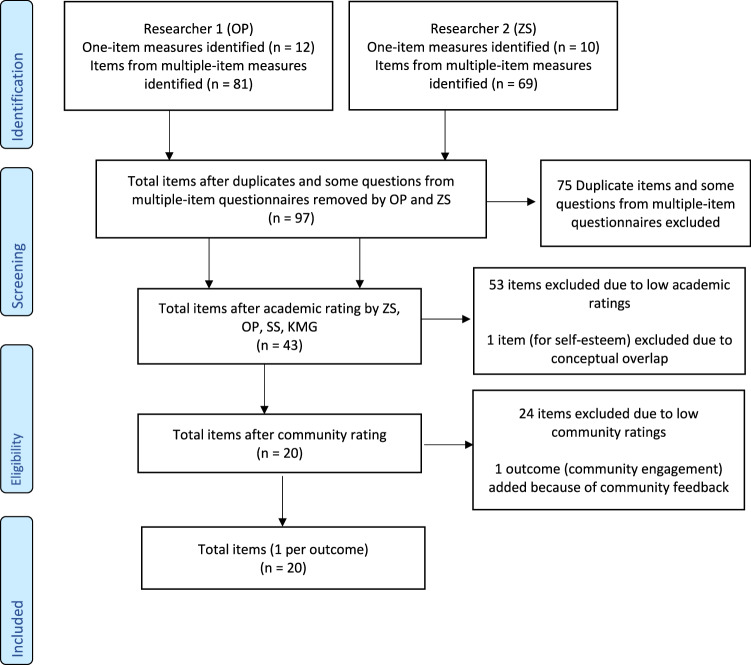
PRISMA-inspired flow chart for item Identification.

Four researchers (SS, KMG, OP, ZS) separately reviewed and rated the 97 items based on the conceptual alignment with the respective outcomes and their definitions, using a four-point scale ranging from 0 (Does not match the definition) to 3 (Greatly matches the definition). When multiple items were rated as “3” for the same outcome, the researchers indicated with an asterisk the item they felt was the best conceptual match. The four researchers then summed the rating scores and met to select the top two items for each outcome. Next, four community-based partners (CM, TC, HF, SC) and two researcher partners (HG, VN) used the same scale and procedures to rate the remaining items. The item that received the highest sum score was kept for each outcome. There were instances when the items received the same sum score for one outcome or there were inconsistent ratings across the team members (CM, TC, HF, SC, HG, VN). The team had two online meetings to discuss and decide on best matches of items to outcomes. The team also identified potential outcomes that were conceptually overlapping or not relevant. As a result, the team removed any overlapping item(s) and made minor modifications to the wording to fit the goal of the SCI Peer Support Evaluation Tool (see Fig. 2 for flow chart). Through the team meetings and follow-up email exchanges, we created a preliminary version of the SCI Peer Support Evaluation Tool consisting of the remaining outcomes and items.

#### Step 4. Generic recommendations on the selection of outcome measurement instruments

We consulted with Executive Directors/Chief Executive Officers (CEOs) and Peer Support Program Coordinators from ten Canadian provincial community-based SCI organizations to discuss the preliminary version of the SCI Peer Support Evaluation Tool. The Directors/CEOs and Coordinators provided feedback, via an online survey, on the relevancy, appropriateness of language use, clarity, specificity/unambiguity, and unintended adverse effects of each item [Supplemental 3]. They also participated in a 2-h online consultation meeting. To modify any item(s) that received a lower rating, participants discussed in breakout rooms and large group discussions. Our team then met to modify the items for each outcome to ensure content validity.

**Fig. 3:**
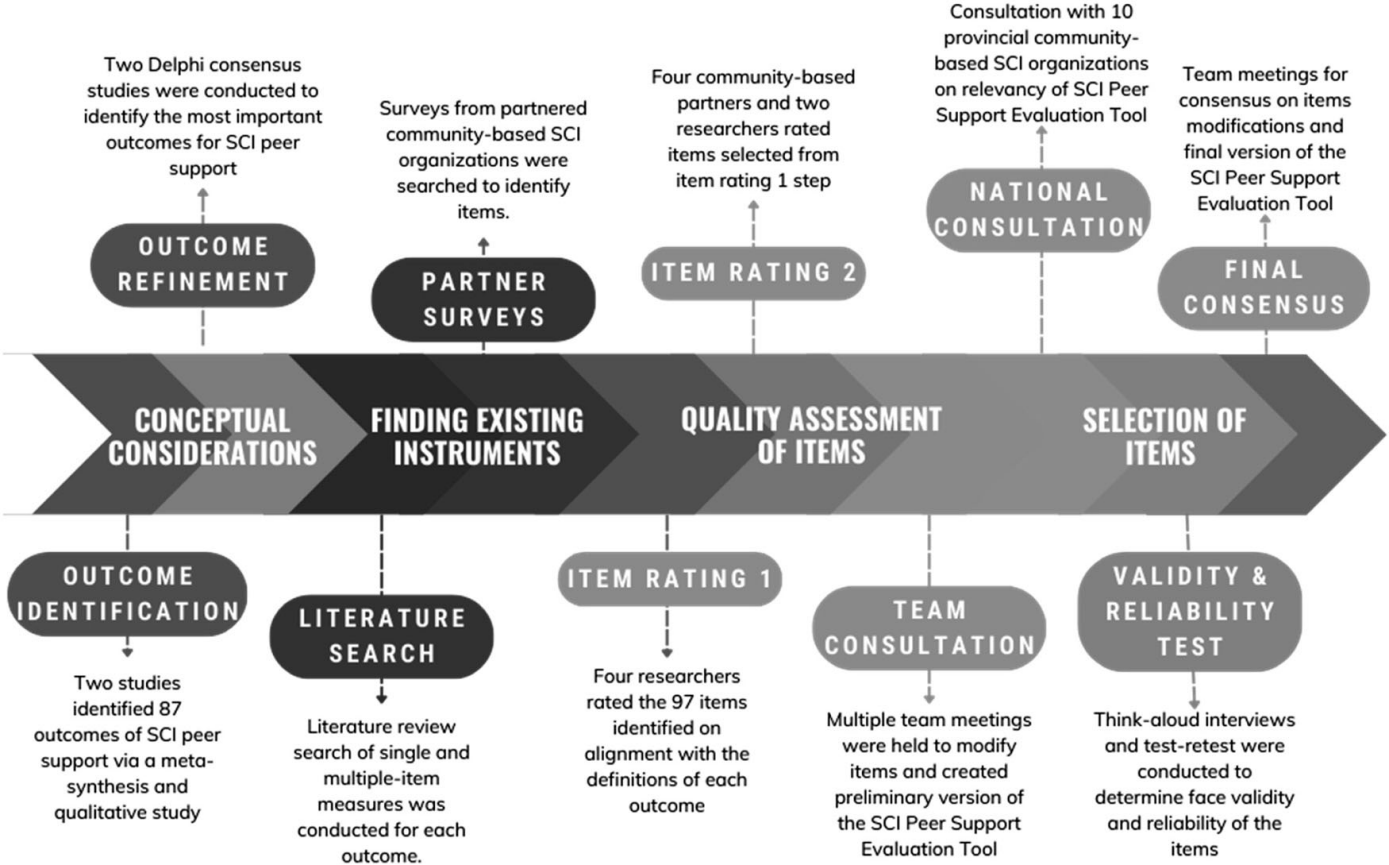
SCI peer support evaluation tool development procedures.

Next, we assessed the response face validity and test-retest reliability of the evaluation tool items based on recent recommendations [17]. For face validity, we used a think-aloud method [18] and for test-retest reliability, we used a 10-day recall testing period. Adults with SCI who received peer support from the partnered community-based SCI organizations were recruited (SCI BC, SCI Saskatchewan, SCI Ontario, Ability New Brunswick). Participants first responded to the items with a researcher present on an online meeting. The researcher prompted participants to voice their thoughts to capture their understanding and approach to answering each item. Once participants answered all the questions, they were asked to reflect on the process of responding to the evaluation tool and provide feedback [Supplemental 4: think-aloud interview guide]. Ten days later, participants were asked to complete the evaluation tool again without thinking aloud. All the interviews were recorded and transcribed. Participants’ utterances for each item from the interviews were pulled from the transcripts. Six researchers (including two people with SCI who were also peer supporters/mentors) (a) rated the correspondence between participants’ responses and outcome definitions, (b) in smaller groups, discussed the main issues (e.g., clarity) and suggested changes for the items with poor correspondence ratings, and (c) met as a larger group to reach consensus on suggested changes prior to bringing forward to the partnership. Reliability of the participants’ responses at two time points was tested using interclass correlation coefficient (ICCs). See Fig. 3 for overview of SCI Peer Support Evaluation development procedures.

## Results

### Step 1. Conceptual considerations

The Delphi studies identified 21 outcomes rated as very important or “one of the most important” for SCI peer support programs. The ratings of each outcome can be found in our previous publication [16].

### Step 2. Finding existing outcome measurement instruments

The online database and community document search yielded a total of 22 single-item measures related to the 21 outcomes identified in Step 1. An additional 150 items from validated multiple-item measures were identified, totaling 172 single item questions. After duplicates and some questions from multiple-item questionnaires were removed (*n* = 75) a total of 97 items remained. During this process, a 22nd outcome was identified (community engagement) and added to the list due to its meaningfulness to the community-based organizations and alignment with the peer support outcome model [14]. Supplemental 5 provides a breakdown of single-and multiple-item measures identified per outcome.

### Step 3. Quality assessment of outcome measurement instruments

Supplemental 6 outlined the 97 items identified in Step 2 and the decisions on retention/removal of each item. During this process, the team agreed on the final 20 outcomes that aligned with our broad conceptualization of peer support outcomes (see Fig. 1) and its respective items. During two partnership meetings, we agreed on using a neutral statement (i.e., “Thinking about my experience with [the peer support program]”) as the stem/preamble for the evaluation tool. Given semantic and agreement scales do not appear to cause more response bias [19] and semantic scales are often referred to item-specific evaluations [20], we also decided to use semantic differential scales with two antonyms at each end of the 5-option scale for the items (e.g., Thinking about my experience with [the peer support program], I feel _____ in my experience with my spinal cord injury/disability: (1) a lot less alone, (2) less alone, (3) as alone as before, (4) more alone, (5) a lot more alone).

### Step 4. Generic recommendations on the selection of outcome measurement instruments

Through consultation with provincial community-based SCI organizations, nine Executive Directors/CEOs and Peer Support Program Coordinators provided feedback (Table 1). All items reached consensus as they received an average rating above 5 out of 7 for relevancy, language appropriateness, clarity, and specificity (Supplemental 7). In the consensus meeting, participants discussed and revised items that may lead to unintended/adverse effects through ratings or comments. For example, we changed the item for Dignity from “My life has _____ value.” To “I feel my sense of self-worth is ______.” due to the low rating and participants’ comments. All modifications were summarized in Supplemental 6.

**Table 1:** Demographic characteristics of participants in step 4.

Variables		Community Consultation (N of participants)	Think Aloud and Test-retest Study^a^ (N of participants)
Eligibility criteria		‐ Have worked as a CEO/director/frontline staff member of a community-based SCI organization that provides peer support programs ‐ Be at least 18 years of age ‐ Understand and communicate in English ‐ Have no cognitive impairments	‐ Have participated in peer support program/service delivered by a community-based SCI organization as a mentee (i.e., someone who receives peer mentorship) ‐ Be at least 18 years of age ‐ Understand and communicate in English ‐ Have no cognitive impairments
Gender	Woman	<5	18
	Man	6	11
	Other/No response	0	<5
Age	18–30	0	<5
	31–65	7	19
	65+	<5	7
Education	High school	<5	5
	College	<5	10
	University	<5	14
	Post-Graduate	<5	<5
Years Worked with Organization	0–2	<5	
	3–4	<5	
	5+	5	
	No response	<5	
Peer Mentor Experience	Yes	8	
	No	<5	
Role in Peer Support	Program Director/CEOs	4	
	Frontline Service Provider	4	
	Other	<5	
Living with SCI	Yes	8	31
	No	<5	
Years Living with SCI	0–9	<5	9
	10–19	<5	9
	20–29	<5	7
	30–39	<5	5
	40–49	<5	<5
	50+	0	0
	No response	<5	0
Level of SCI	Cervical	<5	12
	Thoracic	<5	14
	Lumbar	0	<5
	Sacral	0	0
	No response	<5	<5
Completeness	Complete	<5	12
	Incomplete	<5	17
	No response	<5	<5
Cause of SCI	Traumatic	6	21
	Non-Traumatic	<5	9
	No response	<5	<5
AIS Classification	A	5	9
	B	<5	<5
	C	0	6
	D	0	9
	E	0	<5
	No response/Unknown	<5	<5
Mode of Mobility	Manual Wheelchair	7	14
	Power Wheelchair	<5	8
	Walker	0	5
	Braces	0	0
	Cane	0	0
	Walk Independently	0	<5
	Other/No response	<5	<5
Organizations	SCI BC		14
	SCI Saskatchewan		<5
	SCI Ontario		14
	Ability New Brunswick		<5

^a^20 participants completed both the think-aloud and test-retest components of the study. 11 participants completed only the test-retest component.

Face validity was very good as per participants’ comments during the think aloud interviews. Participants found the items easy to understand and relevant to their peer support experiences. For example, when answering the item for SCI Knowledge, one participant said: “I think I learned a great deal, actually, she [mentor] told me a lot about her experiences with certain things, like sexuality and things like that”. Based on similar responses, no modifications were needed for 14 items. Based on participants’ responses, we deemed wording changes were required for items related to: understanding/feeling understood, normalization, rehabilitation transition skills, dignity, resilience, and coping. For example, when answering the item for Understanding/feeling understood, one participant stated: "I wasn't sure who the people were that the question referred to. Is it the people in the peer program or people outside of SCI? I didn't really know". According to their comment, we changed the item from “I feel there are some people who understand me _____” to “I feel _____ understood”. In examining reliability, the ICC between the test-retest across items ranged between 0.32 and 0.89. However, after identifying and removing outlier scores (*n* =1 to 2 participants for 7 outcomes), ICCs ranged between 0.61 and 0.89 (Supplemental 8), representing an acceptable test-retest reliability. Some of the low ICCs were related to outcomes requiring wording changes (e.g., confidence and belief in oneself).

The research team then created first iterations of changes to the 6 items and presented the changes to the larger partnership in two consensus meetings and a final voting consensus exercise. After these consensus meetings, modifications to the normalization, coping, dignity, and resilience items consisted of minor wording changes for clarity purposes (see Supplemental 8). For the understanding and rehabilitation transition skill outcomes, the items were modified to improve clarity on the intent of the item and enhance the alignment with the outcome (see Supplemental 8). To focus participants’ attention on peer support, we included the stem for each item in the final version. To help participants recall their whole peer support experience, an introductory text was added upon peer review and was approved by the full partnership. This final version, consisting of single items for 20 outcomes, was approved by the full partnership team as having evidence to support its content validity in SCI (Table 2).

**Table 2:** Final version of the SCI peer support evaluation tool.

Preamble	
The following questions are intended to get your perception of the general impact of the [peer support program]. When answering these questions, please think about your whole experience with this program from your first to most recent recalled interaction with members of the [peer support program].	

Outcome (Definition)	Spinal cord injury peer support evaluation tool
Understanding/Feeling Understood (Sharing with someone who "hears me" and "gets what I am saying")	Thinking about my experience with the [Peer Support Program], I feel _____ understood. • A lot less • Less • As • Somewhat Better • Better
(Reduced) Isolation (Feeling (less) lonely)	Thinking about my experience with the [Peer Support Program], I feel _____ in my experience with my spinal cord injury/disability. • A lot less alone • Less alone • As alone • More alone • A lot more alone
Normalization (Having the knowledge that others have had similar experiences)	Thinking about my experience with the [Peer Support Program], I am ______ to see that other individuals with spinal cord injury/disability have experienced similar difficulties/challenges. • Much less likely • Less likely • As likely • More likely • Much more likely
Community Engagement (Integration and participation in the community)	Thinking about my experience with the [Peer Support Program], I participate in programs, activities, or events in the community ______. • Much less often • Less often • As often • More often • Much more often
SCI Knowledge (Having new information and an understanding of living with SCI)	Thinking about my experience with the [Peer Support Program], I learned ______ about spinal cord injury from my peer supporter/mentor. • Nothing • Very little • Something • Quite a bit • A great deal
Rehab Transition Skills (Learning tips and tricks for transitioning out of rehab (e.g., finding/adapting housing, finding/adapting transportation, etc.))	Thinking about my experience with the [Peer Support Program], I learned ______ from a peer supporter/mentor on how to transition to community living (for example, housing, transportation, etc). • No tips and tricks • Very few tips and tricks • Some tips and tricks • Quite a few tips and tricks • A lot of tips and tricks
Health Skills (Learning tips and tricks for maintaining your health (e.g., managing spasm, skin care, etc.))	Thinking about my experience with the [Peer Support Program], I learned ______ from a peer supporter/ mentor on how to maintain my health. • No tips and tricks • Very few tips and tricks • Some tips and tricks • Quite a few tips and tricks • A lot of tips and tricks
Self-care Skills Learning tips and tricks for self-care (e.g., dressing/undressing, bowel/bladder care, personal grooming, etc.)	Thinking about my experience with the [Peer Support Program], I learned ______ from a peer supporter/mentor on how to do self-care activities (for example, dressing, managing bowel/bladder, grooming...). • No tips and tricks • Very few tips and tricks • Some tips and tricks • Quite a few tips and tricks • A lot of tips and tricks
Independence (One's self-sufficiency)	Thinking about my experience with the [Peer Support Program], I feel ______ independent. • Much less • Less • As • More • Much more
Belief in Oneself (One's belief in their capacity to achieve things in the future)	Thinking about my experience with the [Peer Support Program], I feel ______ to reach my goals in life. • Much less capable • Less capable • As capable • More capable • Much more capable
Confidence (Feeling self-assured about one's own qualities/capabilities)	Thinking about my experience with the [Peer Support Program], I am ______ that I can accomplish most things I set out to do. • Much less confident • Less confident • As confident • More confident • Much more confident
Dignity (A sense of worth in oneself)	Thinking about my experience with the [Peer Support Program], my sense of worth in myself is ______. • Worse • Somewhat worse • About the same • Somewhat better • Better
Resilience (The capacity to recover from difficult situations)	Thinking about my experience with the [Peer Support Program], I am ______ to bounce back quickly from difficulties or setbacks. • Much less likely • Less likely • As likely • More likely • Much more likely
Coping (Having strategies to minimize or tolerate stress)	Thinking about my experience with the [Peer Support Program], I am ______ to cope with the stresses/demands of my spinal cord injury/disability. • Much less able • Less able • As able • More able • Much more able
Positive Attitude (A positive way of thinking or feeling about life)	Thinking about my experience with the [Peer Support Program], I am ______ to look at things in a positive way. • Much less likely • Less likely • As likely • More likely • Much more likely
Perseverance (One's ability to do something despite facing difficulties)	Thinking about my experience with the [Peer Support Program], I am ______ to keep going when problems arise. • Much less capable • Less capable • As capable • More capable • Much more capable
Hope (One's expectations that good things will happen)	Thinking about my experience with the [Peer Support Program], I feel ______ hopeful. • Much less • Less • As • More • Much more
Mental Health (Having feelings about one's psychological state)	Thinking about my experience with the [Peer Support Program], my mental health is ______. • Worse • Somewhat worse • About the same • Somewhat better • Better
Happiness (Feeling pleasure and contentment with life)	Thinking about my experience with the [Peer Support Program], I feel ______ happy. • Much less • Less • As • More • Much more
Quality of life (One's standard of health, comfort, happiness, and overall wellness)	Thinking about my experience with the [Peer Support Program], my quality of life is ______. • Worse • Somewhat worse • About the same • Somewhat better • Better

A higher rating on an item indicates more positive evaluation of their peer support experience on the outcome.

**Glossary of Terms**

○ SCI peer support: a peer interaction that aims to help individuals who share similar lived experiences adapt and/or thrive.

○ SCI peer mentorship: a purposeful and unidirectional peer support relationship, where a mentor shares their lived experience with a mentee.

○ Peer supporter/mentor: someone who acts as a source of knowledge, guidance, and/or wisdom to a peer mentee with SCI.

○ Peer mentee: an individual with a SCI who receives knowledge, guidance and/or wisdom from a peer supporter with SCI.

○ Outcome: something that follows as a result or consequence of SCI peer support.

○ [Peer Support Program]: various types of programs, service, or initiatives provided by community-based organizations to facilitate peer support among individuals with SCI. The term “[Peer Support Program]” in bracket can be replaced by the specific name of an organization’s program.

## Discussion

The SCI Peer Support Evaluation Tool fills an important gap in the current SCI peer support literature and practice. For one, there have been issues in comparing the impact of programs within and across organizations due to the difficulties in aggregating data from different SCI peer support programs [7]. This tool provides valid and reliable single-item measures of 20 outcomes that will improve consistency in evaluating SCI peer support programs within (e.g., virtual vs in-person peer support programs) and across organizations in Canada. Similarly, the tool should help reduce the inconsistency of outcome reporting and variation of measurement instruments across the international SCI peer support literature [8]. The single-item approach facilitates the evaluation of peer support programs delivered by community-based organizations or researchers by having easy to use items. The single items can be used independently as only outcomes that align with goals of peer support programs can be selected. The development of the SCI Peer Support Evaluation Tool extends the COS and OMI development literature for persons with disabilities [21] or chronic diseases [22]. This study provides an example of how to develop a COS and OMI by utilizing the research, SCI lived experience, and community programming expertise of partnership members while following the IKT guiding principles (as outlined in Fig. 3 and Supplemental 1).

An IKT approach enabled us to adopt a pragmatic research paradigm that focuses on solving practical problems [23, 24]. We engaged the individuals involved in peer support programs (organization staff, peer supporter/mentors, peer mentees, etc.) at different stages by utilizing IKT partnership strategies [25]. In reflection, strategies “co-writing grant”, “listening to each other”, “engaging persons with SCI” were of particular importance because they maximized opportunities to share decision-making and expertise. Despite the use of these strategies, the length of time to complete this research was longer than expected, requiring us to cope with challenges in losing and onboarding community partners. As partnership research becomes more common, equipping partnerships on how to manage attrition and on-boarding will be important.

From a practical standpoint, evaluating SCI Peer Support programs using this tool will help identify the strongest and weakest part of a program. Such evaluation may guide community-based organizations on how to optimize SCI peer support delivery. The evaluation tool can also support funding applications by providing evidence-based data on the impact of SCI peer support, to support the sustainability of these programs. While an advantage of the tool is that it can measure 20 different outcomes, we appreciate that not all evaluations will require assessments of all 20 outcomes. A benefit of the SCI Peer Support Evaluation Tool is that community-based organizations or researchers aiming to evaluate a peer support program can have the flexibility to select the items that corresponds to outcomes of the tested peer support program, meaning not all 20 items needed to be added in program evaluation efforts. We therefore encourage evaluators to select the items that are most relevant to their research/program evaluation questions and to administer them without additional modifications. These steps will help to ensure consistent, valid, comparable assessments across studies/evaluations. The tool therefore provides a key starting point to assess these 20 identified outcomes of peer support. It does not preclude organizations and programs to assess other outcomes that may be specific to their peer support program such as wheelchair skills or self-management [26].

Despite the benefits of this SCI Peer Support Evaluation Tool, we cannot assume that this tool will automatically be implemented within community-based organizations. Community-based organizations highlighted in our meetings and in past research [7] that they often do not have the knowledge or expertise to use measurement or evaluation tools. However, evidence-based resources are more likely to be adopted by target users if implementation tools are created [27, 28]. Our team is concurrently developing a toolkit to support the implementation of the SCI Peer Support Evaluation Tool. This toolkit will help users to set-up an internal process to identify and select the outcomes and items that are most relevant with their program (visit www.mcgill.ca/scipm for resources). It will also provide decision-making aids for administrating the tool, managing the data, and interpreting the results. Such approaches should then facilitate the use and ultimately lead to greater evaluation of SCI peer support program for SCI community-based organizations.

We acknowledge that single-item measures have their limitations. From a psychometric perspective, multiple items provide better estimates of reliability and capture more information on the social, cognitive, or emotions factors of human experiences [17]. However, community-based organizations have little resources for research-level evaluations, and their evaluations cannot cause high assessment burden to their members. Indeed, it would be nearly impossible, in the SCI peer support context, to assess 20 different outcomes using standard, research-level measures [7, 14]. Using single-item measures is therefore a method to counter participant burden within community contexts [17]. The SCI Peer Support Evaluation Tool provides an evidence-based resource to measure key outcomes within peer support programs delivered by SCI community-based organizations.

Assessing a variety of outcomes with single items, community-based organizations and researchers may better capture the complexity of SCI peer support. In our previous research, we measured only a few outcomes and we concluded that the results did not necessarily reflect the breadth or depth of impact of peer support [29–31]. Assessing multiple outcomes, while minimizing participant burden, might help to elucidate the impact of peer support.

### Study limitations

We recognize that convergent or predictive validity was not assessed, but rather focused on face validity through a think aloud survey [17]. There is now an opportunity to revisit the psychometrics of this tool once it is implemented and used in the community context to examine other dimensions of validity and reliability with a larger sample. These future studies could also examine the relationship and interplay between these outcomes whereby identifying whether peer support has direct or indirect impacts on certain outcomes. Finally, the development of this evaluation tool was conducted in a community-based Canadian context. We acknowledge that SCI peer support programs in Canada primarily adopt a discussion-based approach, while alternative approaches (e.g., activity-based) are also used in and outside Canada. Due to the differences in peer support programs, different outcomes may be identified as important in other contexts. The decision to focus on the Canadian community-based organizations was driven by the goal to address the unique practical need of these organizations in assessing the impact of their programs. Gathering perspectives from individuals involved in diverse peer support programs beyond the Canadian context could offer unique insights in future research. However, given the rigorous approach to develop items for each outcome, the potential for international usage of this evaluation tool remains high. Further validation and refinement of the tool (e.g., updated definition based on international consensus on a peer support taxonomy) with input from a diverse, international perspective would increase its usage beyond the Canadian context.

## Conclusion

The SCI Peer Support Evaluation Tool provides an important and practical resource to facilitate the assessment and evaluation of the complex nature of SCI peer support. The implementation of this tool should facilitate the optimization of peer support programs for people with SCI in Canada, and hopefully serve useful for other international contexts.

## Supplementary information

Supplemental Materials

## Data Availability

Additional data is available from the corresponding author on reasonable request.
