# Supplementary material for: The spinal cord injury (SCI) peer support evaluation tool: the development of a tool to assess outcomes of peer support programs within SCI community-based organizations
Source: Spinal Cord. 2024 Sep 23;62(12):690–9. doi: 10.1038/s41393-024-01033-1 (PMC11621013; doi:10.1038/s41393-024-01033-1)
Supplement: Supplementary file 1 — Supplemental Materials [file 41393_2024_1033_MOESM1_ESM.pdf]

Supplemental 1: Integrated Knowledge Translation Process

Research Stage	Type of Activity	Activity Description	IKT Guiding Principles
**Conceptual Design (grant)**	Discussion (Phone and video calls; one in person meeting)	The research question and methodology was co-developed by community partners from SCI British Columbia (CM), SCI Alberta (TC), SCI Ontario (SC), and Ability New Brunswick (HF) along with researchers (SS, LS, HG, VN, KMG) prior to co-writing a funding grant. The research question and methodology was confirmed at an in-person team meeting. Community partners and researchers were assigned to specific studies within the larger grant.	• partner develop and maintain relationship based on trust, respect, dignity, and transparency<br>• partners share in decision-making<br>• partners foster open, honest, and responsive communication<br>• partners recognize, value, and share their diverse expertise and knowledge<br>• partners are flexible and receptive in tailoring the research approach to match the aims and context of the project<br>• partners can meaningfully benefit by participating in the partnership
**Conceptual Design (item selection process)**	Discussion (Video Calls)	Community partners (CM) and researchers (SS, KMG) were co-leads on deciding the methodologies and process to identify potential items and evaluation of the items.	• partners share in decision-making<br>• partners foster open, honest, and responsive communication<br>• partners recognize, value, and share their diverse expertise and knowledge<br>• partners are flexible and receptive in tailoring the research approach to match the aims and context of the project<br>• partners respect the practical considerations and financial constraints of all partners

Research Stage	Type of Activity	Activity Description	IKT Guiding Principles
**Items decision process**	Discussion (Video Calls and emails)	Community partners (CM, HF, SC, TC) and researchers (SS, KMG, HG, OP, ZS, VN) met to make decisions on the selection of items after the ratings and community consultation phases.	• partners share in decision-making<br>• partners foster open, honest, and responsive communication<br>• partners recognize, value, and share their diverse expertise and knowledge
**Conceptual Design (think aloud)**	Discussion (Video Calls)	Previous (CM, HF, SC) and new (LS – SCI Saskatchewan) community partners and researchers (SS, KMG, HG, OP, ZS, VN) discussed the need and value of conducting this study. The structure and design was co-constructed over two meetings. One community partner (TC) retired from the community organization and thus was not involved in this step.	• partners can meaningfully benefit by participating in the partnership<br>• partners address ethical considerations<br>• partners respect the practical considerations and financial constraints of all partners
**Recruitment (Think aloud)**	Developing & distributing recruitment materials (emails)	SS, ZS, and OP created templates for recruitment emails, consent forms, and interview guides.<br><br>CM, HF, SC, and LS reviewed, approved, and distributed recruitment materials within their organizations.	• partners share in decision-making<br>• partners foster open, honest, and responsive communication<br>• partners recognize, value, and share their diverse expertise and knowledge
**Data Collection (Think aloud)**	Conducting think aloud interviews	OP, ZS, JC, and RS scheduled, conducted, and recorded all interviews. They coordinated the recruitment efforts with all community partners, including reminders and updates on recruitment numbers.	• partners foster open, honest, and responsive communication<br>• partners respect the practical considerations and financial constraints of all partners

Research Stage	Type of Activity	Activity Description	IKT Guiding Principles
**Data Analysis and Interpretation (think aloud)**	Meeting in person	OP, ZS, JC, RS, and SS overviewed all results, created summaries of results, and drafted suggestions for consensus meetings with the larger partnership team.	• partners foster open, honest, and responsive communication
**Final consensus meetings**	Discussion and consensus (Video Calls)	SS, JC, LW prepared and planned the consensus meeting. CM, LS and new members of community partners (JM, RW, S-AW, KF) and researchers (SS, KMG, HG, OP, ZS, VN, LW) participated in the meeting as well as a voted on final changes to the evaluation tool.	• partners share in decision-making<br>• partners foster open, honest, and responsive communication<br>• partners recognize, value, and share their diverse expertise and knowledge<br>• partners can meaningfully benefit by participating in the partnership
**Manuscript Preparation**	E-mail	SS, RS, OP, and ZS led the writing of the manuscript. KMG provided initial round of feedback. All co-authors reviewed the manuscript, provided feedback and approved final content prior to submission for publication.	• partners share in decision-making<br>• partners foster open, honest, and responsive communication<br>• partners recognize, value, and share their diverse expertise and knowledge
**Knowledge Dissemination**	Creating knowledge dissemination tools	SS, LW, OP, and ZS will be creating graphics and infographics for the study results. These tools will be reviewed by researchers and community partners who will form a knowledge dissemination working group.	• partners share in decision-making<br>• partners foster open, honest, and responsive communication<br>• partners recognize, value, and share their diverse expertise and knowledge<br>• partners are flexible and receptive in tailoring the research approach to match the aims and context of the project

Note: Underlined sections in the table are strategies identified in Hoekstra, F., SCI Guiding Principles Consensus Panel, Gainforth, H.L. (2021). Principles and related strategies for spinal cord injury research partnership approaches: a qualitative study. Evidence & Policy, 18, 776-788. DOI: 10.1332/174426421X16161715996124.

CM, TC, SC, HF, and LS are/were directors of spinal cord injury/disability community-based organizations providing years of experience in developing and managing SCI peer support programs.
SS, HG, VN, and KMG are researchers within the spinal cord and disability context who provide expertise in peer support, measurement, and integrated knowledge translations/partnership.
OP, ZS, and LW are graduate students working at different phases of the research phases.
JC (research assistant) and RS (researcher) provided their expertise from their research experience, as people living with SCI, and individuals who have peer support experiences.
JM, RW, S-AW, KF are member of community-based organizations directly working in peer support programs to which some also provided their expertise in living with SCI.

Supplemental 2: Database searching strategy

Outcome		OR		OR
Confidence	AND	Single-item<br>1-item<br>One-item	AND	Measur*<br>Evaluat*<br>Assess*<br>Questionnaire*<br>Survey<br>Scale
		OR		OR
Perseverance	AND	Single-item<br>1-item<br>One-item	AND	Measur*<br>Evaluat*<br>Assess*<br>Questionnaire*<br>Survey<br>Scale
		OR		OR
Independence	AND	Single-item<br>1-item<br>One-item	AND	Measur*<br>Evaluat*<br>Assess*<br>Questionnaire*<br>Survey<br>Scale

[Same set of searching strategy for the other 17 outcomes]

Supplemental 3: Survey

For each item on this survey, the wording [the peer support program/service name] is meant to be replaced with the specific name of your peer support program/service. You can use the name of your program/service when reading the questions if it helps with readability.

Thinking about your SCI peer support program/service, please rate each item measurement in terms of relevance, appropriate language use, specificity/unambiguity, clear expression, and unintended adverse effects. Please read through each item measurement carefully and rate them on these four criteria on a scale of 1 (strongly disagree) to 7 (strongly agree).

Note: You are rating the item measurement and not the outcome itself. For example, *Understanding* is the outcome whereas its respective item measurement which you are to rate is:

Thinking about my experience with [the peer support program/service name], I feel there are some people who understand me ____.

- *A lot less*
- *Less*
- *As much as before*
- *More*
- *A lot More*

Confidence: Thinking about my experience with [the peer support program/service name], I am ______ that I can accomplish most things I set out to do.

- Much less confident
- Less confident
- As confident
- More confident
- Much more confident

	Strongly Disagree	Disagree	Somewhat Disagree	Neither Agree or Disagree	Somewhat Agree	Agree	Strongly Agree	No answer
The item is relevant for SCI organizations.								
The item uses language that is appropriate for SCI organizations.								
The item is clearly expressed.								

The item is specific and unambiguous.								
The item could lead to unintended adverse effects or negative feelings/emotions in respondents.								

[Same set of questions for the other 19 items]

Supplemental 4 - Think Aloud Interview Guide Part 1

Agenda:

□ Overview of study purpose (emphasize to answer the questions within the context of the SCI support/mentorship program)

□ Overview of procedures for the interview

- Concurrent portion (10-20 min)
- Retrospective portion (20-30 min)

□ Check that participant has signed the consent document

□ Turn on Microsoft Teams recording

□ Warm-up: "How many windows do you have in your home?" (2 min)

□ Warm-up: Try out reading the first item on the evaluation tool and think aloud (2 min)

Concurrent Session:

□ Ask the participant to describe how long they have been living with a SCI and how long they received peer mentoring (to provide some context)

□ Each participant completes measure independently while verbalizing their thoughts. Explain to them they should voice through what they think of the question and response formats, how they interpret the questions, the clarity of the questions, and why they chose their answers (and anything else that comes up). Anything that comes up is relevant so keep talking aloud.

- Give a reminder "please keep thinking aloud" or "please keep talking" when is silent for more than ten second.

Retrospective Session:

Objective: To discuss all elements of the evaluation tool (i.e., instructions, response format, and each item)

1. What was your general thoughts and feeling about answering the survey/evaluation tool?
2. Use the following questions to probe each item:
   A. Did the answer choices include your answer?
   B. Did you understand how to answer the question?
   C. Was the wording of the question clear to you?
   D. Is there anything else you want to add about this question?

Concluding Questions:

3. Would you like to add any other information related to the measure we are developing?
4. Do you have any other comments or questions?
5. Schedule the second interview.

Think Aloud Interview Guide Part 2

Agenda:

□ Overview of procedures for this meeting

□ Turn on audio recorder

□ Each participant completes measure independently without verbalizing their thoughts

Key Questions:

Thank you for taking the time to fill out our evaluation tool a second time. I have a few questions I wanted to follow-up with:

1. How was answering the items the second time on your own?
2. Do you have any other comments on the evaluation tool?
   a. Probe for clarity, relevance to SCI peer support, likelihood of completing a sub-set of items if sent by the community organization.
3. Do you have any other comments or questions?

Supplemental 5: Single- and Multiple- Item Measures Identified in the Literature Search per Outcome

Outcome	Single-Item Measures	Multiple-Item Measures	Total Items from Initial Search	Total Items after Duplicates Removed
Normalization	0	4	4	4
SCI Knowledge	1	3	4	4
Independence	1	0	1	1
Confidence	1	3	4	2
Self-esteem	1	4	4	4
Belief in Oneself	0	6	6	3
Reduced Isolation	5	6	11	4
Dignity	0	22	22	12
Perseverance	0	15	15	3
Resilience	0	12	12	5
Health Skills	1	1	1	2
Hope	0	8	8	5
Positive Attitude	0	13	13	8
Happiness	3	11	14	4
Quality of Life	3	2	5	4
Coping	2	7	9	2
Well-Being	2	10	12	6
Positive Mental Health	2	6	8	3
Rehab Transition Skills	0	2	2	2
Understanding	0	5	5	5
Self-Care Skills	0	2	2	2
Community Engagement	0	8	8	8
**Total**	**22**	**150**	**172**	**97**

Supplemental 6: 97 items decision-making process

**Outcome** (Definition)	Item	Resource	Academic rating	Community partners rating	Partnership Meeting & Community Consultation
**Normalization** (Having the knowledge that others have had similar experiences)	"When things are going badly for me, I see the difficulties as part of life that everyone goes through."	Self-Compassion Scale (26-Items) / (Neff, 2003)	Keep	To discuss	Modified to “I am ______ to see my difficulties as a part of life that many others with a spinal cord injury go through.”
	"When I'm down and out, I remind myself that there are lots of other people in the world feeling like I am."	Self-Compassion Scale (26-Items) / (Neff, 2003)	Keep	To discuss	Remove
	"I try to see my failings as part of the human condition"	Self-Compassion Scale (26-Items) / (Neff, 2003)	Remove		
	"When I feel inadequate in some way, I try to remind myself that feelings of inadequacy are shared by most people."	Self-Compassion Scale (26-Items) / (Neff, 2003)	Remove		
**Understanding** (Sharing with someone who	"I felt understood"	NIH - General Life Satisfaction	Keep	Remove	

"hears me" and "gets what I am saying")	"I have someone who understands my problems"	NIH - Emotional Support	Remove		
	"I have someone who will listen to me when I need to talk"	NIH - Emotional Support	Remove		
	"I feel there are people who really understand me"	PROMIS - Emotional Support	Keep	Keep	Keep
	"To what extent have you experienced understanding and support from others, whether emotional or physical?"	Perceived Social Support	Remove		
**Reduced Isolation** (Feeling [less] lonely)	I felt lonely."	Loneliness (Single-item) / (Luchetti et al, 2020)	Remove		
	"I feel less alone in my experience of disability"	Community Organization (AB) (Single-Item)	Keep	Keep	Keep
	"Do you feel lonely?"	Loneliness (Single-item) / (Pynnonen, 2018)	Keep	Remove	
	"In the past week, how often did you feel isolated from others"	COVID-19 Study (Single-Item)	Remove		

**Independence** (One's self-sufficiency)	"I am more independent"	Community Organization (SCI-AB), Single-Item	Keep	Keep	Keep
Self-esteem (A positive view of one's worth or abilities)	"I have high self-esteem"	Single-Item Self-Esteem Scale / (Robins et al., 2001)	Remove		
	"How satisfied are you with yourself?"	Single-Item for Global Self-Esteem (Atroszko et al., 2017)	Keep	To discuss	N/A (Outcome was removed)
	"I felt useful"	NIH - General Life Satisfaction	Remove		
	"I liked my self"	NIH - General Life Satisfaction	Keep	To discuss	N/A (Outcome was removed)
Belief in Oneself (One's belief in their capacity to achieve things in the future)	"It is easy for me to stick to my aims and accomplish my goals"	PROMIS - General Self-Efficacy	Keep	Remove	
	"I can reach my goals in life"	PROMIS - Meaning and Purpose	Keep	Keep	Modified to "I feel ______ to reach my goals in life."
	"My future looks good"	PROMIS - Positive Affect	Remove		
Confidence (Feeling self-assured about	"I felt confident"	NIH - General Life Satisfaction	Keep	To discuss	Remove

one's own qualities/capabilities)	"I believe I am a confident person"	PROMIS - Psychological Illness Impact	Remove		
	"I am confident that I can perform effectively on many different tasks."	NIH - General Life Satisfaction	Keep	To discuss	Remove
	"I can accomplish most things I set out to do."	Moorong Self-Efficacy Scale (Middleton et al., 2003)	Keep	To discuss	Modified to "I am ______ that I can accomplish most things I set out to do."
Dignity (A sense of worth in oneself)	"To what extent do you feel the things you have been doing in your life during the past week are worthwhile"	COVID-19 Study (Single-Item)	Remove		
	"I understand my life's meaning"	NIH - General Life Satisfaction	Remove		
	"My life has a clear sense of purpose"	NIH - General Life Satisfaction	Remove		
	"I generally feel that what I do in my life is valuable and worthwhile"	NIH - Meaning and Purpose	Remove		
	"To me, the things I do are all worthwhile"	NIH - Meaning and Purpose	Remove		

	"My life has value"	NIH - Meaning and Purpose	Keep	Keep	Modified to "I feel my sense of self-worth is ______."
	"My life had purpose"	Neuro-QOL - Positive Affect & Well-Being	Remove		
	"My life had meaning"	Neuro-QOL - Positive Affect & Well-Being	Remove		
	"My life was worth living"	Neuro-QOL - Positive Affect & Well-Being	Keep	Remove	
	"I felt a sense of purpose in my life"	Neuro-QOL - Positive Affect & Well-Being	Remove		
	"I can make sense of my existence"	NIH - General Life Satisfaction	Remove		
	"I can make sense of my life"	NIH - General Life Satisfaction	Remove		
Perseverance (One's ability to do something despite facing difficulties)	"I can keep going when problems arise"	PROMIS - Psychological Illness Impact	Keep	To discuss	Keep
	"It is easy for me to stick to my aims and accomplish my goals"	PROMIS - Psychological Illness Impact	Remove		

	"I believe I can achieve my goals, even if there are obstacles"	Connor-Davidson Resilience Scale (10-Item)	Keep	To discuss	Remove
Resilience (The capacity to recover from difficult situations)	"I am able to adapt to change"	Connor-Davidson Resilience Scale (2-Item)	Remove		
	"I tend to bounce back after illness or hardship"	Connor-Davidson Resilience Scale (2-Item)	Remove		
	"I tend to bounce back quickly after hard times"	Brief Resilience Scale	Keep	To discuss	Modified to “I am ______ to bounce back quickly after hard times.”
	"I usually come through difficult times with little trouble"	Brief Resilience Scale	Keep	Remove	
	"It does not take me long to recover from a stressful event"	Brief Resilience Scale	Remove		
Coping (Having strategies to minimize or tolerate stress)	"I am able to cope better with the physical and emotional impact of my disability"	Community Organization (AB) (Single-Item)	Keep	Keep	Keep
	“How well are you coping with the stress of your job	Single-Item Coping Measure	Keep	Remove	

	right now?" *Note - wording would have to change to remove "of your job"	(Eddy et al., 2019)			
Hope (One's expectations that good things will happen)	"My future looks good"	PROMIS - Posiitve Affect	Keep	To discuss	Remove
	"I felt hopeless"	Neuro-QOL – Depression	Remove		
	"I felt that I had nothing to look forward to"	Neuro-QOL - Depression	Remove		
	"I felt hopeful"	Neuro-QOL - Positive Affect & Well-Being	Keep	To discuss	Keep
	"Even when things were going badly, I still had hope"	Neuro-QOL - Positive Affect & Well-Being	Remove		
Positive Attitude (A positive way of thinking or feeling about life)	"I felt optimistic"	Neuro-QOL - Positive Affect & Well-Being	Remove		
	"I was able to enjoy life"	Neuro-QOL - Positive Affect & Well-Being	Remove		
	"I could laugh and see the humor in situations"	Neuro-QOL - Positive Affect & Well-Being	Keep	Remove	

	"I look at things in a positive way"	PROMIS - Psychological Illness Impact	Keep	Keep	Modified to “I am ______ to look at things in a positive way.”
	"I am positive about my future"	PROMIS - Psychological Illness Impact	Remove		
	"I am feeling optimistic about life’s challenges"	SOM-7	Remove		
	"Right now, I expect things to work out for the best"	SOM-7	Remove		
	"I am expecting things to turn out well"	SOM-7	Remove		
Positive Mental Health (Having positive feelings about one’s psychological state)	"In general, how would you consider your mental health?"	Single-Item / (Macias et al, 2015; Golenbock et al, 2017)	Keep	To discuss	Modified to “My mental health is ______.”
	"In general, how would you rate your mental health, including your mood and your ability to think?"	Global Health	Keep	To discuss	Remove
	"How often have you been bothered by emotional problems such as feeling	Global Health	Remove		

	anxious, depressed, or irritable?"				
Happiness (Feeling pleasure and contentment with life)	"Do you feel happy in general?"	Single-Item Happiness Measure	Keep	To discuss	Remove
	"How happy are you?"	1-10 Happiness Scale	Keep	To discuss	Remove
	"In the past week, how happy did you feel?"	COVID-19 Study (Single-Item)	Remove		
	"I felt happy"	NIH - General Life Satisfaction	Keep	To discuss	Keep
Quality of Life (One's standard of health, comfort, happiness, and overall wellness)	"I have better quality of life"	Community Organization (AB) (Single-Item)	Remove		
	"How have things been going for you in the past four weeks?"	Wasson, 2019	Remove		
	"Considering all parts of my life - physical, emotional, social, spiritual, and financial - my quality of life in the past two days was…"	SIS / (Cohen et al., 1977)	Keep	To discuss	Modified to "My quality of life is ______."
	"My life situation is excellent"	NIH - General Life Satisfaction	Remove		
Well-Being (One's standard	"Taking everything into consideration,	Single-Item / (Headey,	Remove		

of health, comfort, happiness, and overall wellness)	how would you say you are today?"	Kelley, & Wearing 1993)			
	"In general, how satisfied are you currently with your life on the whole?"	Single-Item / (Hajek et al, 2018)	Keep	To discuss	N/A (Outcome was combined with Quality of Life)
	"I feel a sense of purpose in my life"	NIH - Meaning and Purpose	Remove		
	"My life is fulfilling"	NIH - Meaning and Purpose	Remove		
	"I had a sense of well-being"	NIH - Meaning and Purpose	Remove		
	"I had a good life"	NIH - Meaning and Purpose	Remove		
SCI Knowledge (Having new information and an understanding of living with spinal cord injury)	"I learned something from the person I was introduced to"	Community Organization (AB) (Single-Item)	Keep	Keep	Modified to "I learned ______ about spinal cord injury from my peer supporter/mentor."
	"Rate your lifetime knowledge of [psychiatric disorders]" *Note - wording would have to change to put in SCI"	Knowledge of Psych. Disorders / (Swami et al., 2010)	Remove		
	"What is your self-rated knowledge of COVID-19?" *Note -	COVID-19 Study (Single-Item)	Remove		

	wording would have to change to put in SCI"				
	"I have someone to give me information if I need it"	PROMIS - Informational Support	Remove		
Health Skills (Learning tips and tricks for maintaining your health (e.g., managing spasm, skin care, etc.))	"When all is said and done, I am the person who is responsible for managing my health condition"	Patient Activation Measure / (Hibbard et al., 2004)	Remove		Decided to use the item for Self-Care Skills and Rehab Transition Skills: “I learned ______ from my peer supporter/mentor on how to maintain my health.”
	"Taking an active role in my own health care is the most important factor in determining my health and ability to function"	Patient Activation Measure / (Hibbard et al., 2004)	Remove		
Self-Care Skills (Learning tips and tricks for self-care (e.g., dressing/undressing, bowel/bladder care, personal grooming, etc.))	"I use what I have learned from [my mentor] to do [self-care activities]"	Motivated Strategies for Learning Questionnaire / (Rothans et al., 2010)	Keep	To discuss	Modified to “I learned ______ from my peer supporter/mentor on how to do self-care activities.”
	"When I [conduct a self-care activity], I try to put together the information from [my mentor]"	Motivated Strategies for Learning Questionnaire / (Rothans et al., 2010)	Remove		
	"I have received adequate [tips and	COM-B Hand Hygiene	Keep	To discuss	Remove

	tricks] in [self-care] [from my mentor]."	Behaviour Questionnaire / (Lydon et al, 2019)			
	"I know how to conduct [most self-care activities]"	COM-B Hand Hygiene Behaviour Questionnaire / (Lydon et al, 2019)	Keep	To discuss	Remove
Rehab Transition Skills (Learning tips and tricks for transitioning out of rehab (e.g., finding/adapting housing, finding/adapting transportation, etc.))	"I use what I have learned from [my mentor] to [make transition out of rehab]"	Motivated Strategies for Learning Questionnaire / (Rothans et al., 2010)	Keep	To discuss	Modified to " I learned ______ from my peer supporter/mentor on how to transition from rehabilitation to the community.”
	"When I [make transition out of rehab], I try to put together the information from [my mentor]"	Motivated Strategies for Learning Questionnaire / (Rothans et al., 2010)	Remove		
	"I have received adequate [tips and tricks] for my [transition out of rehab] [from my mentor]."	COM-B Hand Hygiene Behaviour Questionnaire / (Lydon et al, 2019)	Keep	To discuss	Remove
	"I know how to [transit out of rehab]"	COM-B Hand Hygiene	Keep	To discuss	Remove

		Behaviour Questionnaire / (Lydon et al, 2019)			
Community Engagement (Integration and participation in the community)	"I found out have to access other programs or supports in the community."	SCI-BC	Remove		
	"I participate more in programs, activities or events in the community."	SCI-BC	Keep	Keep	Keep
	"I contribute to peer program events"	SCI-BC	Remove		
	"I am more involved in my community."	SCI-AB	Keep	Remove	
	"I also participate in my community in other ways (e.g., other volunteer activities, recreation groups, social events, etc.)	SCI-AB	Remove		
	"Do you participate in accessing services in your community?"	Patient-Perceived Participation in Daily Activities Questionnaire / (Noreau et al., 2013)	Remove		
	"Do you participate in activities and	Patient-Perceived	Remove		

	organizations [in your community]? For example, a social club, spiritual or religious groups."	Participation in Daily Activities Questionnaire / (Noreau et al., 2013)			
	"Do you participate in carrying-out civic responsibilities? For example, voting or participating in community meetings and activities."	Patient-Perceived Participation in Daily Activities Questionnaire / (Noreau et al., 2013)	Remove		

Supplemental 7: Community Consultation Survey Results

**Outcome**: Item	Rele-vancy	Lan-guage Appropri ateness	Clarity	Specificity/ Ambiguity	Unintended/ Adverse Effect**	Comments
**Normalization*:** Thinking about my experience with [the peer support program/ service name], I am _______ to see my difficulties as a part of life that many others with a spinal cord injury go through.<br>• Much less likely<br>• Less likely<br>• As likely<br>• More likely<br>• Much more likely	6.22	5.77	5.55	5.33	2.55	The last part of the sentence could be slightly hard to wrap your head around. Maybe instead of "I am __ to see my difficulties as part of life go through" it could say something like "I am __ to see that I am not alone due to the challenges (or obstacles) I face due to my SCI."
**Understanding*:** Thinking about my experience with [the peer support program/ service name], I feel there are some people who understand me ____.<br>• A lot less<br>• Less<br>• As much as before<br>• More<br>• A lot more	6.11	6	5.22	5	2.25	Replace "understand me" with "understand my situation" or "understand how I feel/what I'm going through"

**Reduced Isolation: Thinking about my experience with [the peer support program/service name], I feel _____ in my experience with my disability**<br>• A lot less alone<br>• Less alone<br>• As much as before<br>• More alone<br>• A lot more alone	6.33	6.33	6.44	6.33	2	
**Independence: Thinking about my experience with [the peer support program/service name], I am ____.**<br>• Much less independent<br>• Less independent<br>• As independent<br>• More independent<br>• Much more independent	6.44	6.44	6.44	6.11	2.11	
**Belief in Oneself*:** Thinking about my experience with [the peer support program/ service name], I feel ____ to reach my goals in life.<br>• Much less capable<br>• Less capable<br>• As capable<br>• More capable<br>• Much more capable	5.66	6	5.66	5.44	2.37	

**Confidence: Thinking about my experience with [the peer support program/service name], I am ______ that I can accomplish most things I set out to do.**<br>• Much less confident<br>• Less confident<br>• As confident<br>• More confident<br>• Much more confident	6.44	6.22	6.22	6	2	
**Dignity***: Thinking about my experience with [the peer support program/ service name], I feel my life has _____.<br>• Much less value<br>• Less value<br>• As much value<br>• More value<br>• Much more value	5.33	5.22	5.55	5	4.25	Value can mean different things for different people. For someone who thinks outside the box, this may make them re-evaluate themselves and define value as things like productivity in society, ability to perform certain tasks, earning potential. It's difficult to tie it into a peer relationship.
**Perseverance: Thinking about my experience with [the peer support program/service name], I am ____ to keep going when problems arise.**<br>• Much less capable<br>• Less capable<br>• As capable<br>• More capable<br>• Much more capable	5.77	5.66	5.66	5.66	2.37	

**Resilience: Thinking about my experience with [the peer support program/service name], I am ____ to bounce back quickly after hard times.**<br>• Much less likely<br>• Less likely<br>• As likely<br>• More likely<br>• Much more likely	5.66	5.66	5.77	5.66	2.55	
**Coping: Thinking about my experience with [the peer support program/service name], I am _____ to cope with the physical and emotional impact of my disability.**<br>• Much less able<br>• Less able<br>• As able<br>• More able<br>• Much more able	6.22	6.11	6	5.88	2.55	
**Hope: Thinking about my experience with [the peer support program/service name], I feel ____.**<br>• Much less hopeful<br>• Less hopeful<br>• As hopeful<br>• More hopeful	5.66	5.55	5.77	5.33	2.88	

• Much more hopeful						
**Positive Attitude: Thinking about my experience with [the peer support program/service name], I am ____ to look at things in a positive way.**<br>• Much less likely<br>• Less likely<br>• As likely<br>• More likely<br>• Much more likely	5.77	5.88	6.11	5.62	3.25	
**Positive Mental Health***: Thinking about my experience with [the peer support program/ service name], my mental health, including my mood and my ability to think is ____.<br>• Much worse<br>• Somewhat worse<br>• About the same<br>• Somewhat better<br>• Much better	5.66	5.66	5.44	5.12	3.55	Unsure about the ability to think part. People may see this as referring to cognitive decline, and not necessarily a mental health issue.

**Happiness: Thinking about my experience with [the peer support program/service name], I feel ____.**<br>• Much less happy<br>• Less happy<br>• As happy<br>• More happy<br>• Much more happy	5.88	5.77	5.66	5.55	2.12	
**Quality of Life: Thinking about my experience with [the peer support program/service name] and considering all parts of my life - physical, emotional, social, spiritual, and financial - my quality of life is _____.**<br>• Much worse<br>• Somewhat worse<br>• About the same<br>• Somewhat better<br>• Much better	6.22	5.77	6	5.55	3.33	

**SCI Knowledge: Thinking about my experience with [the peer support program/service name], I learned ______ about spinal cord injury from my peer supporter/mentor.**<br>• Nothing<br>• Very little<br>• Something<br>• Quite a bit<br>• A great deal	6.44	6.33	6	5.88	2.22	
**Health Skills: Thinking about my experience with [the peer support program/service name], I learned ________ from peer supporter/mentor on how to maintain my health.**<br>• No tips and tricks<br>• Very few tips and tricks<br>• Some tips and tricks<br>• Quite a few tips and tricks<br>• A lot of tips and tricks	6.55	6.44	6.22	6.11	2	
**Rehab Transition Skills: Thinking about my experience with [the peer support program/service name], I learned ________ from peer supporter/mentor on how to transition from rehabilitation to the community.**<br>• No tips and tricks<br>• Very few tips and tricks	6.44	6.33	6.22	6.11	1.87	

• Some tips and tricks<br>• Quite a few tips and tricks<br>• A lot of tips and tricks						
**Community Engagement: Thinking about my experience with [the peer support program/service name], I participate in programs, activities or events in the community ____.**<br>• Much less often<br>• Less often<br>• As much as before<br>• More often<br>• Much more often	6.33	6.33	6.11	5.88	2.25	

Notes. *Outcomes discussed in the consultation meeting. **The score for unintended/adverse effect ranges from 1 to 7. A higher score represents the item is more likely to cause unintended/adverse effect. The outcome of **Self-Care Skills (**Thinking about my experience with [the peer support program/ service name], I learned _____ from peer supporter/mentor on how to do self-care activities: No tips and tricks; Very few tips and tricks; Some tips and tricks; Quite a bit of tips and tricks; A great deal of tips and tricks) was accidently omitted from this assessment.

Supplemental 8: Consensus meetings, think aloud, test-retest reliability results

For added clarity and focus on peer support, all questions should begin: Thinking about my experience with the [Peer Support Program]

	**Outcome (Definition)**	**Original**	**Proposed changes to items for partnership consensus meetings**	**Summary of comments from think aloud**	**Test-retest Reliability (interclass correlation)***	**Final consensus meeting results**
1	Understanding<br><br>(Sharing with someone who "hears me" and "gets what I am saying")	I feel there are some people who understand me ______.<br><br>• A lot less<br>• Less<br>• As much as before<br>• More<br>• A lot more	Thinking about my experience with the [Peer Support Program], I feel _____ understood.<br>• A lot less<br>• Less<br>• As<br>• Somewhat Better<br>• Better	To eliminate risk of confusion about who is being referred to as "some people", wording was changed. This new item is our second option during the item selection process. We feel this item responds to participants' concerns.	0.714 (n = 24);<br><br>Moderate reliability	Agreed on changes to the item for the think aloud.

2	(Reduced) Isolation<br><br>(Feeling (less) lonely)	I feel _____ in my experience with my disability.<br><br>• A lot less alone<br>• Less alone<br>• As alone as before<br>• More alone<br>• A lot more alone	Thinking about my experience with the [Peer Support Program], I feel _____ in my experience with my disability.<br><br>• A lot less alone<br>• Less alone<br>• As alone<br>• More alone<br>• A lot more alone	The response "as alone as before" was changed to "as alone" to simplify the question and align with response options across questions.	0.321 (n = 25)<br><br>After removing outliers: 0.859 (n = 23)<br><br>Good reliability	Added disability after spinal cord injury.
3	Normalization<br><br>(Having the knowledge that others have had similar experiences)	I am ______ to see that many others with spinal cord injury have experienced similar difficulties/challenges.<br><br>• Much less likely<br>• Less likely<br>• As likely<br>• More likely<br>• Much more likely	Thinking about my experience with the [Peer Support Program], I am ______ to see that others with spinal cord injury have experienced similar difficulties/challenges.<br><br>• Much less likely<br>• Less likely<br>• As likely<br>• More likely<br>• Much more likely	The word "many" was removed from the question given participants deliberated too long on the how many should they consider.	0.776<br><br>Good reliability	Added disability after spinal cord injury.
4	Community Engagement<br><br>(Integration and participation in the community)	I participate in programs, activities, or events in the community ______.<br><br>• Much less often<br>• Less often	Thinking about my experience with the [Peer Support Program], I participate in programs, activities, or events in the community ______.	A few comments referring to the time frame in question, such as before program, or as	0.546 (n = 23)<br><br>After removing outliers:	No change

		• As much as before<br>• More often<br>• Much more often	• Much less often<br>• Less often<br>• As often<br>• More often<br>• Much more often	a result of program. We believe this will be resolved having the stem before each question.	0.802 (n = 22)<br><br>Good reliability	
5	SCI Knowledge<br><br>(Having new information and an understanding of living with SCI)	I learned ______ about spinal cord injury from my peer supporter/mentor.<br><br>• Nothing<br>• Very little<br>• Something<br>• Quite a bit<br>• A great deal	No changes to the item		0.893 (n = 25)<br><br>Good reliability	No change
6	Rehab Transition Skills<br><br>(Learning tips and tricks for transitioning out of rehab (e.g., finding/adapting housing, finding/adapting transportation, etc.)	I learned ______ from peer supporter/mentor on how to transition from rehabilitation to the community.<br><br>• No tips and tricks<br>• Very few tips and tricks<br>• Some tips and tricks<br>• Quite a few tips and tricks<br>• A lot of tips and tricks	Thinking about my experience with the [Peer Support Program], I learned ______ from peer supporter/mentor on how to transition from rehabilitation to the community.<br><br>• No tips and tricks<br>• Very few tips and tricks<br>• Some tips and tricks<br>• Quite a few tips and	The last option in the responses ("I did not participate…") was added as a participant's commented that they did not participant in peer support during rehabilitation and did not know how to	0.855 (n = 23)<br><br>Good reliability	Reword to move focus from the rehabilitation phase to the transition to community phase

			tricks<br>• A lot of tips and tricks<br>• I did not participate in peer support during rehabilitation	answer this question.		
7	Health Skills<br><br>(Learning tips and tricks for maintaining your health (e.g., managing spasm, skin care, etc.))	I learned ______ from peer supporter/ mentor on how to maintain my health.<br><br>• No tips and tricks<br>• Very few tips and tricks<br>• Some tips and tricks<br>• Quite a few tips and tricks<br>• A lot of tips and tricks	No changes to the item		0.866 (n = 24)<br><br>Good reliability	No change
8	Self-care Skills<br><br>Learning tips and tricks for self-care (e.g., dressing/undressing, bowel/bladder care, personal grooming, etc.)	I learned ______ from peer supporter/mentor on how to do self-care activities.<br><br>• No tips and tricks<br>• Very few tips and tricks<br>• Some tips and tricks<br>• Quite a few tips and tricks	Thinking about my experience with the [Peer Support Program], I learned ______ from peer supporter/mentor on how to do self-care activities (for example, dressing, managing bowel/bladder, grooming...).	Some examples of self-care activities were added for clarification.	0.852 (n = 25)<br><br>Good reliability	Agreed on changes to the item for the think aloud.

		• A lot of tips and tricks	• No tips and tricks<br>• Very few tips and tricks<br>• Some tips and tricks<br>• Quite a few tips and tricks<br>• A lot of tips and tricks			
9	Independence<br><br>(One's self-sufficiency)	I am ______.<br><br>• Much less independent<br>• Less independent<br>• As independent<br>• More independent<br>• Much more independent	Thinking about my experience with the [Peer Support Program], I feel ______ independent.<br><br>• Much less<br>• Less<br>• As<br>• More<br>• Much more	Added independent to the item as participants mentioned wanting to know the topic/idea of the question prior to reading the response options.	0.391 (n = 24)<br><br>After removing outliers: 0.784 (n = 22)<br><br>Good reliability	No change
10	Belief in Oneself<br><br>(One's belief in their capacity to achieve things in the future)	I feel ______ to reach my goals in life.<br><br>• Much less capable<br>• Less capable<br>• As capable<br>• More capable<br>• Much more capable	No changes to the item		0.548 (n = 23)<br><br>After removing outliers: 0.771 (n = 21)<br><br>Good reliability	No change

11	Confidence<br><br>(Feeling self-assured about one's own qualities/capabilities)	I am ______ that I can accomplish most things I set out to do.<br><br>• Much less confident<br>• Less confident<br>• As confident<br>• More confident<br>• Much more confident	No changes to the item		0.552 (n = 22)<br><br>No outliers identified.<br><br>Moderate reliability	No change
12	Dignity<br><br>(A sense of worth in oneself)	I feel my sense of self-worth is ______.<br><br>• Worse<br>• Somewhat worse<br>• About the same<br>• Somewhat better<br>• Better	Thinking about my experience with the [Peer Support Program], my sense of worth in myself is ______.<br><br>• Worse<br>• Somewhat worse<br>• About the same<br>• Somewhat better<br>• Better	Adjustment in the question wording for clarity, especially surrounding the word self-worth.	0.570 (n = 24)<br><br>After removing outliers: 0.772 (n = 23)<br><br>Good reliability	Agreed on changes to the item for the think aloud.
13	Resilience<br><br>(The capacity to recover from difficult situations)	I am ______ to bounce back quickly after hard times.<br><br>• Much less likely<br>• Less likely<br>• As likely<br>• More likely<br>• Much more likely	Thinking about my experience with the [Peer Support Program], I am ______ to bounce back quickly from difficult situations.<br><br>• Much less likely<br>• Less likely<br>• As likely	The use of the term 'hard times' was seen to refer to longer periods, whereas 'difficult situations' is	0.530 (n = 25)<br><br>After removing outliers: 0.767 (n = 23)<br><br>Good reliability	Modified to "to bounce back quickly from difficulties or setbacks." to provide the situational context as per our definition.

			• More likely<br>• Much more likely	more situational.<br><br>The wording “difficult situations” come from our definition of resilience.		
14	Coping<br><br>(Having strategies to minimize or tolerate stress)	I am ______ to cope with the physical and emotional impact of my disability.<br><br>• Much less able<br>• Less able<br>• As able<br>• More able<br>• Much more able	Thinking about my experience with the [Peer Support Program], I am ______ to cope with the demands of my disability.<br><br>• Much less able<br>• Less able<br>• As able<br>• More able<br>• Much more able	The term 'physical and emotional impact' was seen by many as too restrictive. Using 'the demands of my disability' should open up responses	0.491 (n = 24)<br><br>After removing outliers: 0.656 (n = 23)<br><br>Moderate reliability	Modified to “the stresses/demands” to provide a psychological component.
15	Positive Attitude<br><br>(A positive way of thinking or feeling about life)	I am ______ to look at things in a positive way.<br><br>• Much less likely<br>• Less likely<br>• As likely<br>• More likely<br>• Much more likely	No changes to the item		0.627 (n=25)<br><br>Moderate reliability	No change

16	Perseverance<br><br>(One's ability to do something despite facing difficulties)	I am ______ to keep going when problems arise.<br><br>• Much less capable<br>• Less capable<br>• As capable<br>• More capable<br>• Much more capable	No changes to the item		0.692 (n=24)<br><br>Moderate reliability	No change
17	Hope<br><br>(One's expectations that good things will happen)	I feel ______.<br><br>• Much less hopeful<br>• Less hopeful<br>• As hopeful<br>• More hopeful<br>• Much more hopeful	Thinking about my experience with the [Peer Support Program], I feel ______ hopeful.<br><br>• Much less<br>• Less<br>• As<br>• More<br>• Much more	Added hopeful to the item as participants mentioned wanting to know the topic/idea of the question prior to reading the response options.	0.631 (n=24)<br><br>Moderate reliability	Agreed on changes to the item for the think aloud.
18	Positive Mental Health<br><br>(Having positive feelings about one's psychological state)	My mental health is ______.<br><br>• Worse<br>• Somewhat worse<br>• About the same<br>• Somewhat better<br>• Better	No changes to the item		0.784 (n=23)<br><br>Good reliability	No change

19	Happiness<br><br>(Feeling pleasure and contentment with life)	I feel ______.<br><br>• Much less happy<br>• Less happy<br>• As happy<br>• More happy<br>• Much more happy	Thinking about my experience with the [Peer Support Program], I feel ______ happy.<br><br>• Much less<br>• Less<br>• As<br>• More<br>• Much more	There were numerous comments about the time period being considered as well as 'in relation to?' Including the stem is very important.<br><br>Added happy to the item as participants mentioned wanting to know the topic/idea of the question prior to reading the response options.	0.756 (n=24)<br><br>Good reliability	Agreed on changes to the item for the think aloud.
20	Quality of life<br><br>(One's standard of health, comfort, happiness, and overall wellness)	My quality of life is ______.<br><br>• Worse<br>• Somewhat worse<br>• About the same<br>• Somewhat better<br>• Better	No changes to the item		0.610 (n=24)<br><br>Moderate reliability	No change

*Note: Moderate reliability (0.5~0.75), good reliability (0.75~0.9), excellent reliability (above 0.9). For some of the outcomes, we reran the analyses after removing outlying case(s) with a ≥3 value difference at 2 times.
